# Risk of hepatitis C virus related hepatocellular carcinoma between subjects with spontaneous and treatment-induced viral clearance

**DOI:** 10.18632/oncotarget.14937

**Published:** 2017-02-01

**Authors:** Chung-Feng Huang, Ming-Lun Yeh, Ching-I Huang, Yu-Ju Lin, Pei-Chien Tsai, Zu-Yau Lin, Soa-Yu Chan, Shinn-Cherng Chen, Hwai-I Yang, Jee-Fu Huang, Sheng-Nan Lu, Chia-Yen Dai, Chin-Lan Jen, Yong Yuan, Gilbert L’Italien, Li-Yu Wang, Mei-Hsuan Lee, Ming-Lung Yu, Wan-Long Chuang, Chien-Jen Chen

**Affiliations:** ^1^ Hepatobiliary Division, Department of Internal Medicine, Kaohsiung Medical University Hospital, Kaohsiung Medical University, Kaohsiung, Taiwan; ^2^ Faculty of Internal Medicine, School of Medicine, College of Medicine, Kaohsiung Medical University, Kaohsiung, Taiwan; ^3^ Institute of Clinical Medicine, National Yang-Ming University, Taipei, Taiwan; ^4^ The Genomics Research Center, Academia Sinica, Taipei, Taiwan; ^5^ The Division of Hepato-Gastroenterology, Department of Internal Medicine, Kaohsiung Chang Gung Memorial Hospital, Kaohsiung, Taiwan; ^6^ Chang Gung University School of Medicine, Kaohsiung, Taiwan; ^7^ The Global Health Economics and Outcomes Research, Bristol-Myers Squibb, Princeton, NJ, United States of America; ^8^ The Yale University School of Medicine, New Haven, CT, United States of America; ^9^ MacKay Medical College, Taipei, Taiwan; ^10^ Institute of Biomedical Sciences, National Sun Yat-Sen University, Kaohsiung, Taiwan; ^11^ Academia Sinica, Taipei, Taiwan; ^12^ Liver Center, Division of Gastroenterology, Massachusetts General Hospital, Harvard Medical School, Boston, MA, United States of America

**Keywords:** HCV, HCC, spontaneous clearance, treatment

## Abstract

**Background/Aims:**

Both spontaneous hepatitis C virus (HCV) clearance and the achievement of sustained virological response (SVR) by anti-viral therapy greatly reduce the incidence of hepatocellular carcinoma (HCC). The current study aimed to compare the risk of HCC between the two patient groups

**Methods:**

A total of 313 subjects with spontaneous HCV clearance (SC) and 564 age- and sex-matched patients in the treatment-induced SVR group were enrolled for analysis.

**Results:**

Nineteen (2.2%) of the 877 patients developed HCC during 6,963 person-years of follow-up. Fourteen (2.5%) SVR patients and 5 (1.6%) SC patients developed HCC (P=0.004). Cox regression analysis of factors predictive of HCC included SVR (versus SC: hazard ratio [HR]/ 95% confidence interval [CI]: 5.83/1.27-26.88), diabetes (HR/CI:3.41/1.21-9.58), and age (HR/CI: 1.07/1.01-1.14). Of the 564 SVR patients, eleven (5.9%) of the 187 patients with fibrosis stage 2-4 (F2-4) and 2 (0.9%) of the 226 patients with F01 developed HCC (P=0.01). Compared to SC subjects, only SVR patients with F2-4 (P<0.001) but not F0-1(P=0.60) had a higher risk of HCC development. Cox-regression analysis using liver fibrosis as a variable demonstrated that factors associated with HCC included SVR with F2-4 (versus SC: HR/CI: 10.06/2.20-45.98), diabetes (HR/CI:3.23/1.14-9.19), and age (HR/CI: 1.08 1.02-1.15).

**Conclusions:**

Compared to subjects with spontaneous viral clearance, subjects with antiviral treatment-induced HCV viral clearance remain at high risk for HCC development, especially if they have significant hepatic fibrosis. These results may provide important information for decision-making regarding the prioritization of current direct antiviral agents in resource-limited countries.

## INTRODUCTION

Spontaneous hepatitis C virus (HCV) seroclearance occurs in 20-30% of patients after acute infection [1–3]. Subjects with spontaneous HCV seroclearance have been at a much lower risk of HCC development compared to those with persistent HCV viremia [4]. Chronic HCV infection has become one of the leading causes of hepatocellular carcinoma (HCC). It is estimated that a global population of 185 million people are infected with HCV [5], leading to the increasing incidence of HCV-related HCC worldwide [6]. During the past decades, the development of successful antiviral therapy (e.g., achievement of sustained virological responses, SVR) with interferon-based therapy could attain a sustainable HCV seroclearance for years [7], significantly reducing the risk of cirrhosis and HCC development [8–10]. Nevertheless, it is unclear if treatment-induced HCV seroclearance achieved by SVR could decrease the incidence of HCC to a similar rate observed in subjects with self-limited HCV infections. We herein addressed the issue by conducting an age and sex-matched case-control study using a clinical cohort of chronic hepatitis C (CHC) patients with successful antiviral therapy and a community cohort of patients with spontaneous HCV seroclearance.

## RESULTS

### Patient characteristics

A total of 313 subjects in the SC group (participants who had anti-HCV seropositivity and undetectable HCV RNA at baseline (n = 291) and those who had anti-HCV seropositivity and detectable HCV RNA levels at baseline, but HCV RNA became undetectable after more than one-year follow-up (n = 22)) and 564 age- and sex-matched patients in the SVR group were enrolled for analysis with a follow-up period of 4,224and 2,739 person-years, respectively. The baseline characteristics are shown in Table 1. SVR patients had higher serum levels of aspartate aminotransferase (AST) and alanine aminotransferase (ALT), a lower proportion of favorable IL-28B genotype carriage, and a higher proportion of diabetes, smoking and alcohol consumption. 11.5% of the SVR patients and 6.7% of the SC patients had elevated transaminases. Compared to patients with normal liver function, those with elevated transaminases had a higher proportion of diabetes (18.4% vs. 8.5%, P=0.03).

**Table 1:** Baseline characteristics in patients with spontaneous or treatment-induced HCV clearance

	Treatment-inducedHCV clearance(SVR, n=564)	SpontaneousHCV clearance(n=313)	P value
Age, n (%)			
30-39	77 (13.7)	54 (17.3)	0.30
40-49	127 (22.5)	79 (25.2)	
50-59	238 (42.2)	119 (38.0)	
60-69	122 (21.6)	61 (19.5)	
Sex, n (%)			
Female	360 (63.8)	211 (67.4)	0.29
Male	204 (36.2)	102 (32.6)	
AST (U/L), n (%)			
<40	495 (87.8)	285 (91.1)	0.02
40-79	57 (10.1)	15 (4.8)	
>=80	8 (1.42)	6 (1.92)	
Unknown	4 (0.7)	7 (2.2)	
ALT (U/L), n (%)			
<45	495 (87.8)	285 (91.1)	0.04
45-89	52 (9.2)	14 (4.5)	
>90	14 (2.5)	7 (2.2)	
Unknown	3 (0.5)	7 (2.2)	
Fibrosis, n (%)			
F0-1	226 (40.1)	NA	
F2-4	187 (33.2)		
Unknown	151 (26.8)		
IL-28B rs8099917, n (%)			
GG	3 (0.5)	0 (0.0)	0.0002
GT	62 (11.0)	15 (4.8)	
TT	397 (70.4)	273 (87.2)	
Unknown	102 (18.1)	25 (8.0)	
Diabetes, n (%)			
Yes	71 (12.6)	12 (0.6)	<0.0001
No	490 (86.9)	299 (95.5)	
Unknown	3 (0.5)	2 (0.6)	
Cigarette smoking, n (%)			
Yes	134 (23.8)	16 (5.1)	<0.0001
No	427 (75.7)	251 (80.2)	
Unknown	3 (0.5)	46 (14.7)	
Alcohol drinking, n (%)			
Yes	93 (16.5)	3 (1.0)	<0.0001
No	467 (82.8)	295 (94.3)	
Unknown	4 (0.7)	15 (4.8)	

Note: SVR: sustained virological response; AST: aspartate aminotransferase; ALT: alanine aminotransferase; IL-28: interleukin-28B genotype.

### Risk of HCC development among the entire cohort

**Figure 1:**
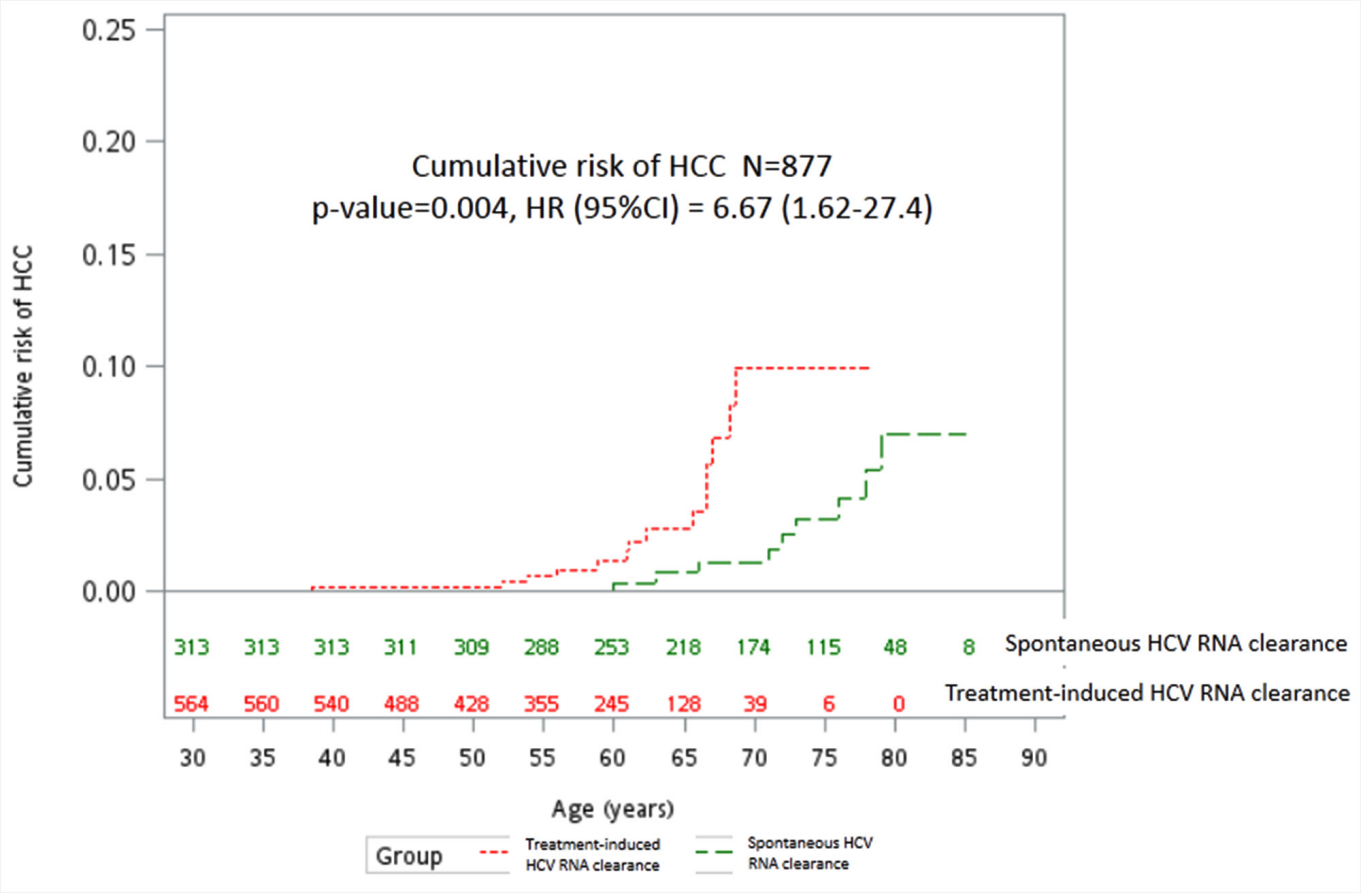
Cumulative risk of HCC between subjects with spontaneous and treatment-induced HCV RNA clearance HCC: hepatocellular carcinoma; HCV: hepatitis C virus; HR: hazard ratio; CI: confidence intervals.

Nineteen (2.2%) of the 877 patients developed HCC over 6,963 person-years of follow-up, including fourteen (2.5%) SVR patients and 5 (1.6%) SC patients with an incidence of 0.51% and 0.12% per person-year, respectively (P=0.004) (Figure 1 and Table 2). In the univariate analysis, patients who developed HCC were older, had a higher proportion of diabetes and were more likely to be in the SVR group (versus SC) (Table 2). Cox regression analysis of the factors predictive of HCC included treatment induced SVR (versus SC: HR/CI: 5.83/1.27-26.88, P= 0.024), diabetes (HR/CI:3.41/1.21-9.58, P=0.02), and age (HR/CI: 1.07/1.01-1.14, P=0.02) (Table 2, model 1).

**Table 2:** Risk factors for HCC development

	All patients(N=877)	HCC cases(n=19)	Crude HR (95% CI)	Adjusted HR (95% CI)Model 1	Adjusted HR (95% CI)Model 2
Age, n (%)					
<55	494	6 (1.2)	1.00 (reference)	1.07 (1.01-1.14)	1.08 (1.02-1.15)
≥55	383	13 (3.4)	3.06 (1.16-8.05)		
Sex, n (%)					
Female	571	11 (1.9)	1.00 (reference)	1.00	1.00
Male	306	8 (2.6)	1.46 (0.59-3.62)	1.19 (0.39-3.65)	1.14 (0.37-3.46)
Diabetics, n (%)					
No	789	13 (1.7)	1.00 (reference)	1.00	1.00
Yes	83	6 (7.2)	6.10 (2.28-16.33)	3.41 (1.21-9.58)	3.23 (1.14-9.19)
Unknown	5	0 (0)	--	--	--
IL-28 rs8099917, n (%)					
TT	670	14 (2.1)	1.00 (reference)		
GG/GT	80	3 (3.8)	2.26 (0.65-7.91)		
Unknown	127	2 (1.6)	0.91 (0.21-4.04)		
Cigarette smoking, n (%)					
No	678	14 (2.1)	1.00 (reference)	1.00	1.00
Yes	150	4 (2.7)	1.83 (0.59-5.64)	1.20 (0.32-4.51)	1.39 (0.38-5.17)
Unknown	49	1 (2.0)			
Alcohol drinking, n (%)					
No	762	16 (2.1)	1.00 (reference)		
Yes	96	3 (3.1)	2.23 (0.63-7.87)		
Unknown	19	0 (0)	--		
Type of HCV RNA clearance, n (%)					
Spontaneous	313	5 (1.6)	1.00 (reference)	1.00	1.00
Treatment-induced	564	14 (2.5)	6.67 (1.62-27.4)	5.83 (1.27-26.9)	
F0-1 (treatment-induced)	226	2 (0.9)	2.66 (0.38-18.6)		1.98 (0.26-14.8)
F2-4 (treatment-induced)	187	11 (5.9)	11.79 (2.77-50.2)		10.06 (2.20-46.0)
Unknown (treatment-induced)	151	1 (0.7)			

Note: HCC: hepatocellular carcinoma; IL-28B: interleukin 28B genotype; HR: hazard ratio; CI: confidence intervals; F0-1: Metavir fibrosis score 0 or 1; F2-4: Metavir fibrosis score 2, 3 or 4.

### Impact of liver fibrosis on HCC development in SVR patients

**Figure 2:**
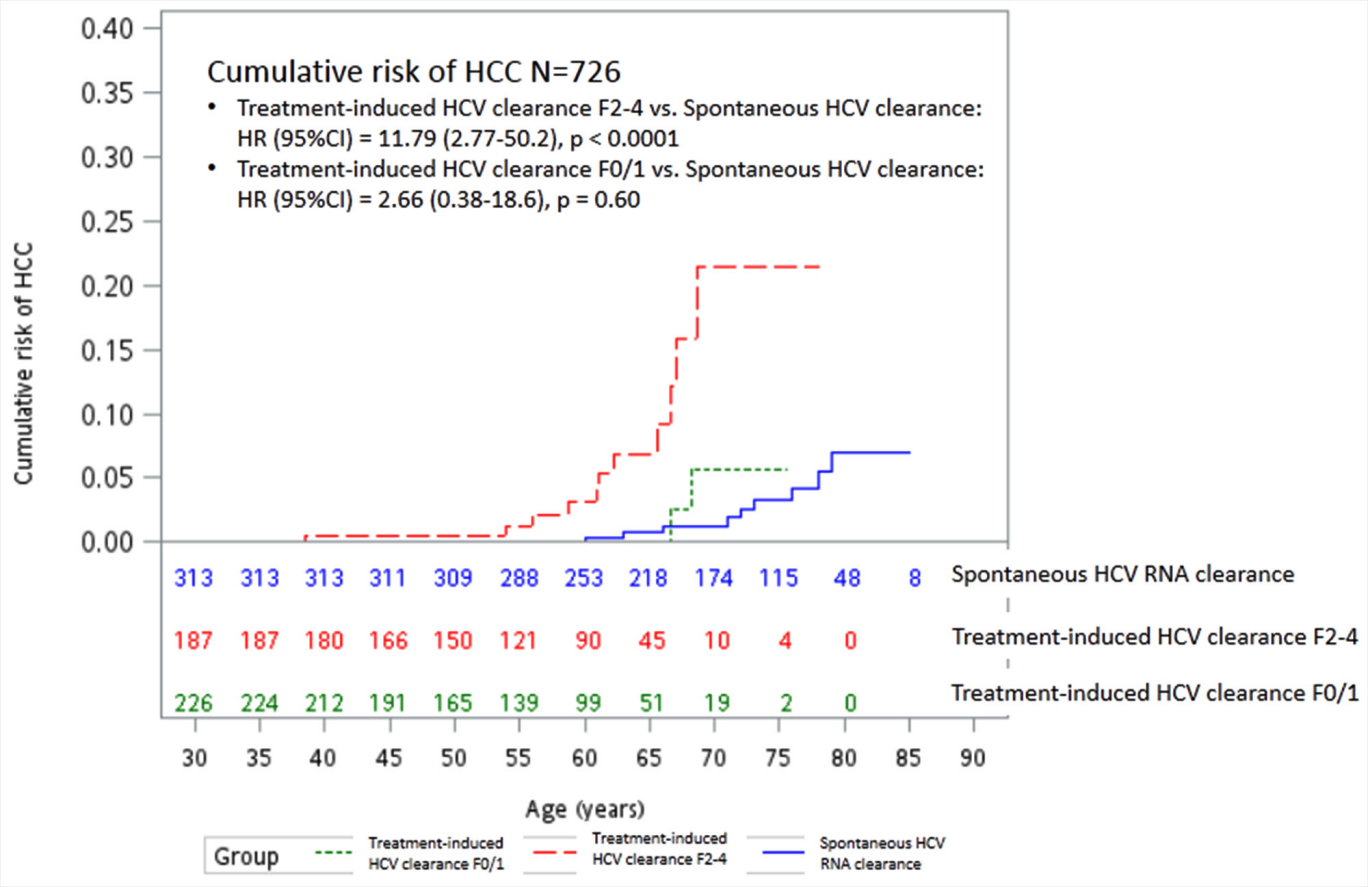
Cumulative risk of HCC between subjects with spontaneous and treatment-induced (Fibrosis score 0 or 1 versus Fibrosis score 2-4) HCV RNA clearance HCC: hepatocellular carcinoma; HCV: hepatitis C virus; HR: hazard ratio; CI: confidence intervals.

Four hundred and thirteen (73.2%) of the 564 SVR patients had liver histology available within six months of initiating the antiviral therapy. Of them, eleven (5.9%) of the 187 patients with F2-4 and two (0.9%) of the 226 patients with F0-1 developed HCC (Log-Rank test P=0.01). Compared to SC subjects, only SVR patients with F2-4 (Log-Rank test P<0.001), but not F0-1(Log-Rank test P=0.60), had significantly higher risk of HCC development (Figure 2 and Table 2, model 2). A Cox-regression analysis using liver fibrosis as a variable demonstrated that factors associated with HCC included SVR with F2-4 (versus SC: HR/CI: 10.06/2.20-45.98, P= 0.003), diabetes (HR/CI:3.23/1.14-9.19, P=0.03), and age (HR/CI: 1.08 1.02-1.15, P=0.015), but did not include SVR with F0-1 (versus SC: HR/CI: 1.98/0.26-14.8).

## DISCUSSION

To date, most of the studies that investigated the beneficial effects of SVR upon HCV-related HCC prevention compared to patients who failed viral eradication or described the differential benefits of antiviral therapy on different SVR subgroups [10]. The magnitude of HCC risk reduction by antiviral treatment has seldom been compared with self-limited HCV infection. Subjects with self-limited HCV infection share common environmental and behavioral risk factors with CHC patients. Selecting subjects with spontaneous HCV clearance as the comparator may have the advantage of eliminating selection bias when evaluating liver-related clinical outcomes [11]. In the current study, we demonstrated that successful viral eradication with antivirals greatly reduced HCV-related HCC occurrence. Nevertheless, the risk remains higher compared to subjects with spontaneous viral clearance, especially among those with pre-existing significant hepatic fibrosis.

Spontaneous seroclearance of HBV DNA and HBsAg are important predictors of reduced HBV-related HCC risk [12]. It has been suggested that HCC occurrence is rare in chronic hepatitis B (CHB) patients who experienced HBsAg seroclearance after nucleoside analogue therapy [13]. On the other hand, compared with inactive CHB carriers, there remains a higher risk for HCC even if the virus was suppressed with oral antiviral therapy [14]. Genetic instability due to HBV viral integration may play a role in the hepatic carcinogenesis. Unlike HBV infection, HCV replication could be completed aborted, which decreases the HCC risk by 75% compared to patients with viral persistence [10]. The results have raised concerns regarding the clinical outcome of treatment-induced SVR compared to self-limited HCV infections. We observed that there remains a six-fold HCC risk for those with successful viral eradication. Notably, the risk was similar to controls in SVR patients without significant fibrosis. The American Association for the Study of Liver Disease has advocated that SVR patients with mild liver disease should receive the same medical care that is recommended for patients who were never infected with HCV [15]. One of the major treatment concerns is prioritization and justification of patients with F2. We have proved that CHC patients with F2 would carried a HCC risk of 6.6 fold as compared to those with F0-1 if they were left with persist viremia [16]. The World Health Organization (WHO) has updated patients with a METAVIR score ≥ 2 as having “significant fibrosis”. Consequently, patients with significant fibrosis had a ten-fold risk of HCC even though they could benefit from successful HCV eradication. Careful monitoring of HCC development remains warranted in these patient groups [16]. Age was an independent risk factor for HCC, which echoes our previous finding that attention should also be paid to elderly patients who achieved an SVR [9]. The issue of the impact of diabetes on HCC is conflicting. A recent meta-analysis has shown that CHC patients carried higher risks of HCC if they possess DM [17]. While the threat decreases with successful HCV eradication, it still remains in diabetic patients. As a consequence, diabetic patients should be viewed as a high priority for treatment [18] with persistent follow-up visits after antiviral therapy.

The role of Il-28B genetic variants in the interferon-based antiviral treatment response is well documented. However, it impact on HCV related HCC remains elusive. We have demonstrated that unfavorable Il-28B genotype, rs8099917 TG/GG genotype, was associated with HCC risk in untreated cohort [3]. On the other hand, we recently demonstrated that it no longer played a role in the post-treatment cohort [19]. In the current study, Il-28B genetic variants also did not determine HCC occurrence in patients with viral clearance. This is in line with the hypothesis that successful viral eradication may overcome the poor genetic predispositions in terms of HCC occurrence. Further studies in different ethnicities are warranted to validate our finding.

One of the major limitations of the current study was that we used patients with self-limited HCV infections as healthy controls. Indeed, the incidence of spontaneous HCV clearance in CHC patients is rare [20]. It has been reported that up to 17% of patients who have self-limited HCV infections cleared the virus after more than 1 year of exposure [21]. Yet However, only 22 (7.56%) of the spontaneous sero-converters in our study had delayed HCV clearance, and the impact of viral persistence on the hosts in terms of liver fibrosis and hepatic carcinogenesis may not be significant. Furthermore, different characteristics between the two groups may interfere with the association with HCC. Spontaneous resolvers are usually younger [11, 20]. It has been recently suggested that that SC in the CHC population possessed four characteristics, younger age, female gender, low HCV RNA levels and HBV coinfection [20]. Among them, age [9], sex and HBV dual infection sex are known as risk factors for HCV related HCC. HBV dual infection was not included in the study. We attempted to adjust the impact of the potential confounders by conducting the age- and sex-matched study. Patients with HBs Ag seropositivity were excluded. However, we failed to exclude subjects with occult HBV infection (OBI) in the current study. The influence of OBI in HCC remains conflicting. Nevertheless, it has been suggested that it has less impact on HCV related HCC [22].

In conclusion, treatment-induced SVR greatly reduces the risk of HCC development. Nevertheless, patients with pre-existing significant hepatic fibrosis remain to be at a higher risk compared to subjects with self-limited HCV infections. All patients who are chronically infected with HCV should be treated, except for individuals with anticipated short-life expectancies [15]. Direct antiviral agents (DAAs) are currently the mainstream of treatment toward CHC. However, it not unaffordable and inaccessible for the majority patients and countries. Treatment at early stage can reduce HCC risk as much as population with spontaneous viral clearance. HCV should be eradicated before the development of significant fibrosis on the host. The results may provide important information for decision-making regarding the prioritization of the current high-cost DAAs for CHC in resource-limited countries.

## MATERIALS AND METHODS

Subjects with spontaneous HCV seroclearance, namely the SC group, were selected from the Risk Evaluation of Viral Load Elevation and Associated Liver Disease/Cancer (R.E.V.E.A.L) HCV cohort. The R.E.V.E.A.L-HCV study is a community-based cohort and enrolled participants from seven townships in Taiwan during 1991-1992. The details of the study participants, interview, blood collection and laboratory examinations have been described previously in detail. [4, 23] Sex- and age- matched CHC patients with treatment-induced SVR after antiviral therapy, namely the SVR group, were consecutively enrolled at a medical center and three regional hospitals between1991 and2011. Patients who had evidence of underlying HCC, hepatitis B virus (defined as HBs antigen seropositivity, Abbott, North Chicago, IL) or human immunodeficiency virus (HIV) co-infections were excluded from both groups. In the SVR group, patients receiving either conventional interferon or peginterferon with or without ribavirin were enrolled if they achieved an SVR, defined as seronegativity of HCV RNA throughout a 24-week post-treatment follow-up period. The subjects with spontaneous HCV RNA seroclearance was defined as participants who had anti-HCV seropositivity and undetectable HCV RNA at baseline and participants who had anti-HCV seropositivity and detectable HCV RNA levels at baseline, but HCV RNA became undetectable after more than one-year follow-up [3]. HCV antibody was detected using third-generation enzyme immunoassay (Abbott Laboratories, North Chicago, IL). Serum levels of HCV RNA in the SC group were measured using the COBASTaqMan HCV test, version 2 (Roche Diagnostics, Indianapolis, NJ, detection limit: 25 IU/mL). In the SVR group, the HCV RNA levels were detected using a qualitative real-time polymerase chain reaction (PCR) (COBAS AMPLICOR Hepatitis C Virus Test, ver. 2.0; Roche, Branchburg, NJ, USA, detection limit: 50 IU/ml) and a quantification branched DNA assay (Versant HCV RNA 3.0, Bayer, Tarrytown, New Jersey, USA; quantification limit: 615 IU/ml). Liver histology, which was obtained within one year of starting antiviral therapy in the SVR group, was graded and staged according to the scoring system described by Scheuer [24]. All of the participants provided their written informed consent for the intervention. This study was approved by the Institutional Review Board of the College of Public Health, National Taiwan University, Taipei, Taiwan and Kaohsiung Medical University Hospital, Kaohsiung, Taiwan, and conformed to the guidelines of the International Conference on Harmonization for Good Clinical Practice.

### Genetic testing

Interleukin 28B (IL-28B) rs8099917 was selected as the single-nucleotide polymorphism (SNP) to be tested for its association with HCC. The genetic testing was determined using methods that have been described previously [3, 25–27].

### Statistical analyses

The baseline characteristics of the clinical cohort and R.E.V.E.A.L.-HCV cohort were compared by chi-squared tests. The cumulative risk of the incidence of HCC among patients who occurred spontaneous HCV RNA clearance (R.E.V.A.E.L.-HCV cohort) and treatment-induced RNA clearance was estimated by Kaplan-Meier method, and the statistical significance of the difference was examined by log-rank tests. Cox proportional hazards models were used to estimate multivariate-adjusted hazard ratios (HR) with 95 percent confidence intervals (95% CI) for HCC risk for various groups with RNA clearance after adjustment for other risk factors. Statistical significance levels were determined by a 2-sided P-value of 0.05. The proportionality assumption (non-changing HRs over time) of Cox models was examined, and the assumption was not violated. All analyses were performed using the SAS statistical software package (release 9.1; SAS Institute Inc., Cary, NC).

## Abbreviations

ALT: alanine aminotransferase
AST: aspartate aminotransferase
CHC: chronic hepatitis C
DAAs: direct antiviral agents
HCC: hepatocellular carcinoma
HCV: hepatitis C virus
HIV: human immunodeficiency virus
IL-28B: interleukin 28B
SVR: sustained virological response
SNP: single-nucleotide polymorphism
SC: seroclearance.
